# The Effect of Glue Cohesive Stiffness on the Elastic Performance of Bent Wood–CFRP Beams

**DOI:** 10.3390/ma13225075

**Published:** 2020-11-11

**Authors:** Bartosz Kawecki, Jerzy Podgórski

**Affiliations:** Faculty of Civil Engineering and Architecture, Lublin University of Technology, ul. Nadbystrzycka 40, 20-618 Lublin, Poland; j.podgorski@pollub.pl

**Keywords:** FEM analysis, glue laminated timber (GLT), wood–CFRP girders, double-lap connection, cohesive stiffness

## Abstract

This paper presents experimental, theoretical and numerical studies of wood-CFRP beams bonded with polyurethane (PUR) adhesive. The analyses include two types of CFRP (carbon fibre-reinforced polymer) strengthening configurations and pure glue laminated timber beams as a reference. Through detailed analyses of a double-lap connection on blocks with and without CFRP strips, the authors state that neglecting the cohesive stiffness of adhesive layers may lead to an overestimation of an overall beam’s stiffness. This is significant with wood–CFRP connections, which showed values two times lower than with wood–wood connections. Theoretical modelling of the equivalent area used in a theory of composites provided much stiffer behaviour of the beams than in laboratory experiments. It proves that a PUR glue eliminates the possibility of using simple models that assume a perfect connection between bonded parts. These conclusions led the authors to use the finite element method (FEM) to take into account the cohesive stiffness. The FEM, based on the properties obtained from a double-lap joint analysis, allowed for the precise prediction of the elastic stiffness of the beams.

## 1. Introduction

Using CFRP (carbon fibre-reinforced polymer) is increasingly popular nowadays. Its popularity is growing because it is light-weight and has high stiffness and strength properties. It is used in civil engineering for strengthening existing structures or elements produced in a factory. Researchers have tried to describe the advantages of its composites by various experimental tests. This introduction has two parts. The first is testing gluing wood–wood and wood–FRP connection, and the second one is testing girders of a full cross-section strengthened or reinforced with FRP (fibre-reinforced polymer) tapes.

### 1.1. Adhesively Bonded Connections

**Figure 1:**
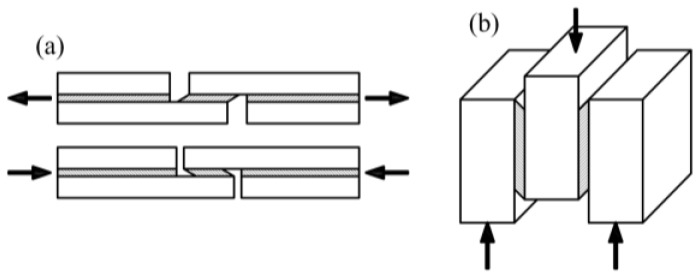
Configurations for testing lap joints in glue laminated timber (GLT—**a**) single-lap joint, (**b**) double-lap joint.

At the beginning, we searched the literature for studies that tested adhesive joints in a wood–wood configuration. Lavisci [1] proposed a method of testing joints in a shear state by applying tension or compression according to the scheme shown in Figure 1a. Gereke [2] carried out a numerical analyses of a similar lap joint in a glue laminated timber (GLT). Almeida [3] and Cavalheiro [4] tested a maximal shearing stress on specimens composed of three elements in a double-lap configuration as shown in Figure 1b.

**Figure 2:**
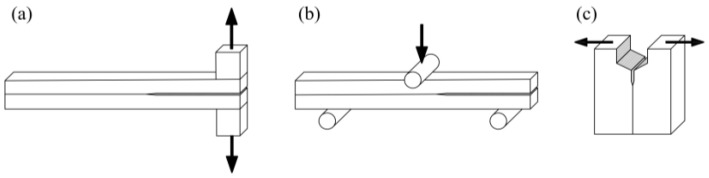
Different methods for testing adhesive connections—(**a**) DCB (double cantilever beam), (**b**) ENF (end notched flexure), (**c**) WS (wedge-splitting).

Wang [5] and Xu [6] researched cracking of GLT on DCB (double cantilever beam) and ENF (end notched flexure) samples (Figure 2a,b). Fortino [7] performed numerical analyses on the delamination of two samples, the first on the WS (wedge-splitting) as shown in Figure 2c and the second on the DCB as shown in Figure 2a.

**Figure 3:**
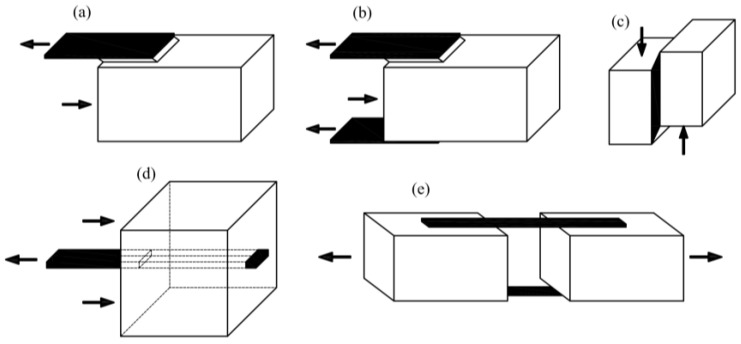
Methods of strengthening solid wood with fibre-reinforced polymer (FRP) outside—(**a**) single-lap joint test, (**b**) double-lap joint test, (**c**) block shear test, (**d**) pull-out test, (**e**) four-lap joint test.

The next case is testing joints in a wood–CFRP configuration. Many authors have studied both single and double-lap joints made of epoxy glue. Vessby [8], Wan [9,10], Biscaia [11], Subhani [12] and Vahedian [13,14,15,16,17] examined the strain’s redistribution in a single-lap joint between the wood and the CFRP plate (Figure 3a). Vessby [8] performed numerical modelling, while Biscaia [11] and Vahedian [14] proposed an analytical solution. Biscaia [18] extended the research with another double-lap scheme (Figure 3b).

Arriaga [19] performed a block shear test (Figure 3c) with GFRP (glass fibre-reinforced polymer), and Sena-Cruz [20] and Fava [21] carried out a pull-out test of two types of composite plates—GFRP and CFRP—glued at varying lengths (Figure 3d). Lee [22] presents tests performed on CFRP strips separated from wood as shown in Figure 3e.

In summary, researchers have used a variety of static patterns to test wood–wood and wood–FRP connections. The lack of their systematisation and designation of the best method confirms the complexity of the problem. Joints in elements containing wood and FRP composites are a current research topic as shown by the up-to-date scientific publications. The most common examined glue is epoxy because of its wide ranging applicability and chemical binding properties. In addition, it does not need the application of high pressure to bond the elements. The authors have found no examples of examining polyurethane (PUR) glue stiffness in the literature.

### 1.2. Strengthening Solid Wood with FRP Strips

In the next step, we searched the literature for methods of strengthening and reinforcing wood with FRP composites. The first method is gluing strips to the bottom of a girder on the entire cross-section width. Researchers prefer this location, because FRP is most efficient under tension. Fiorelli [23] carried out studies on beams reinforced with GFRP and CFRP. Kossakowski [24] used three types of hand-laminated mats: glass, aramid and carbon. Kim [25,26] examined the girders with an incision in the middle of the span. He used different thicknesses of CFRPs with prefabricated strips and a different number of carbon fibre layers with manual lamination. Corradi [27] analysed the uncertainty of the material properties of the girders reinforced with CFRP and GFRP. de Jesus [28] tested two variants of CFRP reinforcement on half and the entire span of the beam by performing three-point bending.

**Figure 4:**
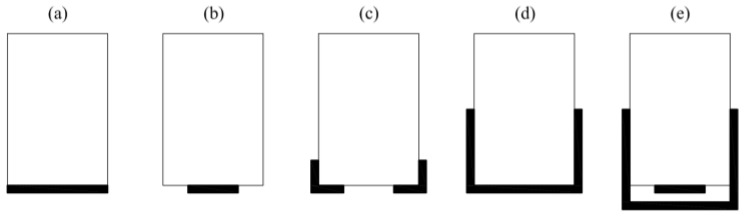
Methods of strengthening solid wood with FRP outside—(**a**) full-width, (**b**) half-width, (**c**) angles, (**d**) U-shaped, (**e**) cumulative: half-width with U-shaped.

The second method of strengthening solid wood consists of gluing the composite to the bottom of the girder but only on the variant width of the cross-section. Andor [29] strengthened the girders using CFRP glued in three variants: one layer on the entire width; two layers on the entire width; one layer on the half of the cross-section width. Borri [30] reinforced half of the beam width with two thicknesses and in the form of angles (Figure 4a–c).

Another way method is to fold the composite onto the vertical planes of the girders. Rosa Garcia [31,32] applied the reinforcement using CFRP and BFRP (basalt fibre-reinforced polymer) in a U-shaped form using three-point bending. Rescalvo [33] carried out a comparison of different configurations of timber beam reinforcement using CFRP in the same scheme and performed a statistical analysis based on a large number of samples. He considered the following reinforcement variants: full-width (Figure 4a); half-width (Figure 4b); U-shaped (Figure 4d); cumulative: half-width with U-shaped (Figure 4e).

**Figure 5:**
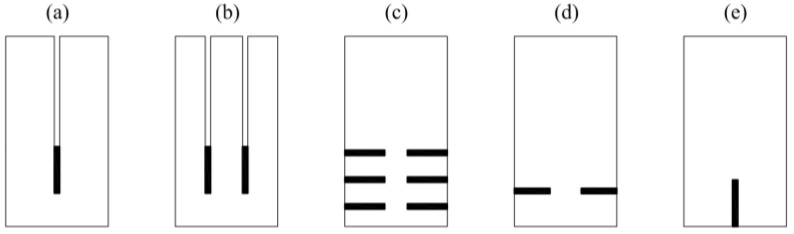
Methods of strengthening solid wood with FRP in incisions—(**a**) one-internal vertical, (**b**) two-internal vertical, (**c**) six-edge horizontal, (**d**) two-edge—horizontal, (**e**) one-edge vertical.

The last method is cutting a cross-section and gluing a plate into the prepared incision. Jankowski [34] and Nowak [35] conducted such studies; Figure 5a–c show these configurations. Further, they simulated cracks by making other incisions. Nowak [35] conducted FE (finite element) modelling on the beams presented in Figure 5a–c. Schober [36] added reinforcements in two other configurations, which are presented in Figure 4b and Figure 5d,e.

**Figure 6:**
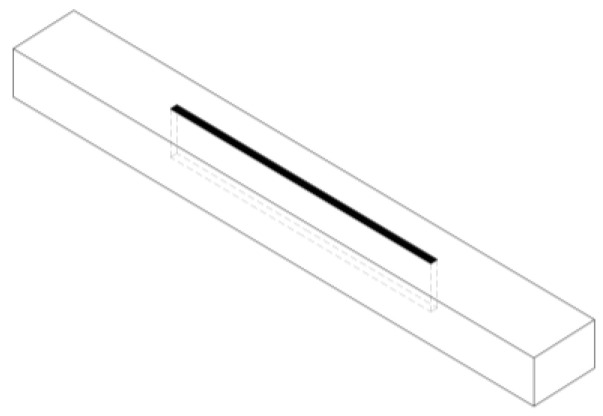
Method proposed in References [37,38].

Morales-Conde [37] proposed a technique for reinforcing wooden beams in the form of vertical strips glued along the entire height of the cross-section (Figure 6). In his research, he used different lengths of reinforced zones. Basterra [38] used a similar solution for glued laminated timber beams composed of two lamellas glued in a vertical scheme.

### 1.3. Strengthening GLT with FRP Strips

The first method is to fix FRP to the bottom of a girder. It is the same solution as solid timber. In most of the works listed below, the researchers used an epoxy resin and the static scheme was four-point bending. 

Nadir [39] analysed a reinforcement made of CFRP and GFRP. He conducted tests on a wood–FRP composite and shear tests of the adhesive. Vahedian [40] considered strengthening CFRP for different thicknesses of the strips. Thornallsson [41] reinforced GLT using GFRP and BFRP. Brunetti [42] tested two different glues and different thicknesses of reinforcements. Here, PUR glue bonding was used for sticking CFRP to wood first time. The last two authors [41,42] conducted a detailed statistical analysis of the results. Glisovic [43,44] tested glued laminated timber reinforced with CFRP in various configurations such as those presented by Schober (Figure 4b and Figure 5d,e). In another work [45], he proposed FE modelling as performed by Nowak [35] and Khelifa [46]. Subhani [47] suggested strengthening laminated beams with CFRP in two variants—at the bottom of the beam and in a U-shape (Figure 4a,d).

**Figure 7:**
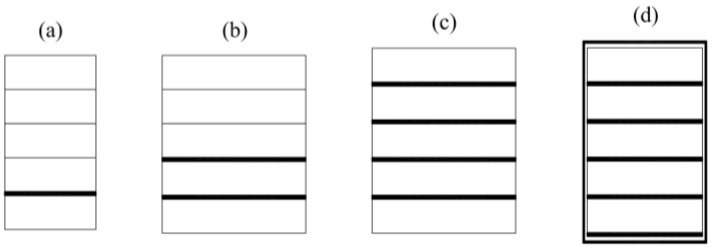
Methods of strengthening solid wood with FRP in different configurations—(**a**) one strip, (**b**) two strips, (**c**) four strips, (**d**) four strips and around the perimeter.

Fiorelli [48] and Raftery [49,50,51] conducted laboratory tests and statistical analyses of the results. Figure 7a shows the configuration of a prepared reinforcement. Then, they change the material of the bonding layer between wood and CFRP from an epoxy resin to a PRF (phenol resorcinol formaldehyde) adhesive. In the latest work, Raftery [50] performed FE modelling based on the assumptions in the papers of Nowak [35], Glisovic [45] and Khelifa [46]. Osmannezhad [52] proposed gluing GFRP on epoxy glue between the layers of GLT in various configurations as shown in Figure 7b,c. He conducted three-point bending tests and statistical analyses of the results. Shi [53] glued GFRP into each joint of GLT and then around the perimeter of the beam cross-section as shown in Figure 7d.

Bal [54] analysed the three-point bending of GLT reinforced with GFRP on PRF glue. The author examined laminated girders in vertical and horizontal schemes (Figure 7c). He attempted to test the shear resistance of the bonding with glass fibre glued in a pattern (Figure 1a). Yang [55] considered the case of girders subjected to four-point bending. He used GFRP and CFRP fibres glued onto PRF glue in several configurations as shown in Figure 4b, Figure 5d,e and Figure 7a–c.

### 1.4. Introduction Summary

The presented publications confirm an increase in the stiffness and general performance of the girders after adding FRP to the cross-section. The number of papers describing PUR adhesives is very low, and the most commonly examined glue was epoxy.

Due to the lack of tests bonding wood–CFRP composites on PUR adhesives, we considered this type of bonding for this paper. It is a very practical approach, because the glue used for GLT in construction is PUR. Developing this research may enable the production of wood–CFRP composites in a factory dedicated to glue laminated timber.

Researchers have published the abovementioned papers in recent years, which makes the research up to date. However, a full recognition of the topic requires other extensive tests and analyses.

The literature survey showed that researchers neglected the glue layer stiffness when modelling the described girders [35,45]. The authors of this paper state that, in case of PUR (soft glue compared to epoxy), including the cohesive stiffness of the connection has a significant influence on bent girders. The experimental, theoretical and numerical analyses presented in this paper confirm these conclusions.

## 2. Preparation of the Composite Samples

The technology required to make glued laminated timber comprises several stages. The first stage is drying the raw material to 12% humidity and rough planing of the parts. Next, the machines find the defects by reducing the strength, such as large knots, resin bags or plugs, that the workers should cut from the material. Then the boards are connected with finger joints. This enables the creation of a lamella of a length exceeding 20 m. The next stage is planing to a thickness of 40 ± 2 mm, applying glue and compressing in a special press. Finally, the finished beams are planed on 4 sides and chamfered.

**Figure 8:**
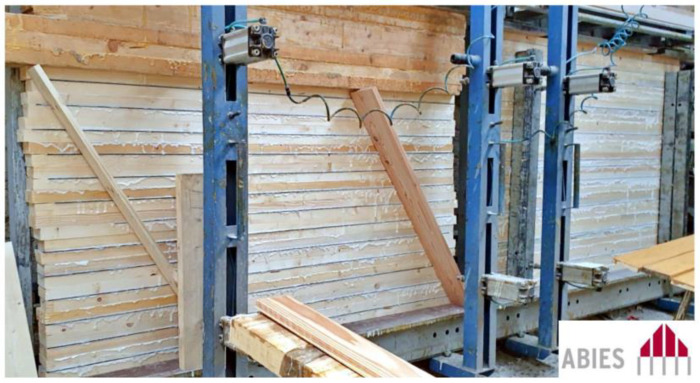
Gluing process at ABIES Poland Ltd.

Figure 8 presents the gluing process at ABIES Poland Ltd. (Pszów, Poland). The advantage of factory production of the specimens over their preparation in laboratory conditions is complying with the technological regime used in producing glued laminated timber.

Preparation of the samples consists of gluing wood lamellas with CFRP tapes on PUR glue. The materials used in this process were C24 class softwood lamellas with the dimensions of 40 mm × 93.4 mm (produced by ABIES Poland Ltd., Pszów, Poland), S&P C-Laminate SM 100/1.4 CFRP tape (produced by S&P Poland Ltd, Malbork, Poland) and Loctite HB 110 Purbond glue (produced by Henkel Poland Ltd., Warsaw, Poland).

**Figure 9:**
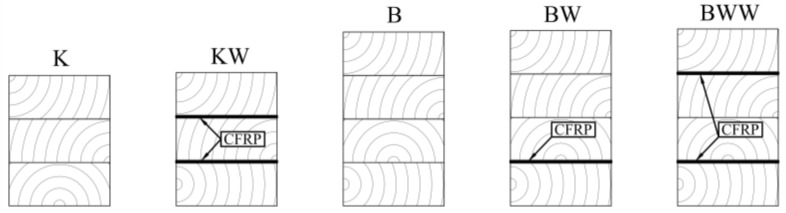
Wood–FRP girders configurations.

The specimens were 2 m long with a rectangular cross-section. The width of each specimen was approximately 93.4 mm and the height depended on the timber lamella and CFRP strip configurations (Figure 9). The beams named “K” and “KW” were going to be cut into pieces for a double-lap shear test.

## 3. Evaluation of the Cohesive Stiffness of a PUR Adhesive

The PUR glue is soft, which results in avoiding the shear stresses concentration in the adhesive layer. However, it causes a reduction in the stiffness of the connections comparing to, for example, epoxy adhesives. Preliminary studies showed that the cohesive stiffness of the glue layers may have a significant effect on the elastic performance of bent wood–CFRP beams. The primary purpose of this section is to describe the method for determining and calculating the properties to apply when modelling with the finite element method (FEM).

Preparation of the specimens comprised cutting the beams “K” and “KW” (Section 2, Figure 9) into 18 small blocks (120 mm × 74 mm × 93.4 mm) for each configuration. Then, the elements had the same connection properties as the full-scale girders. This relates to the actual work of the adhesive joint.

**Figure 10:**
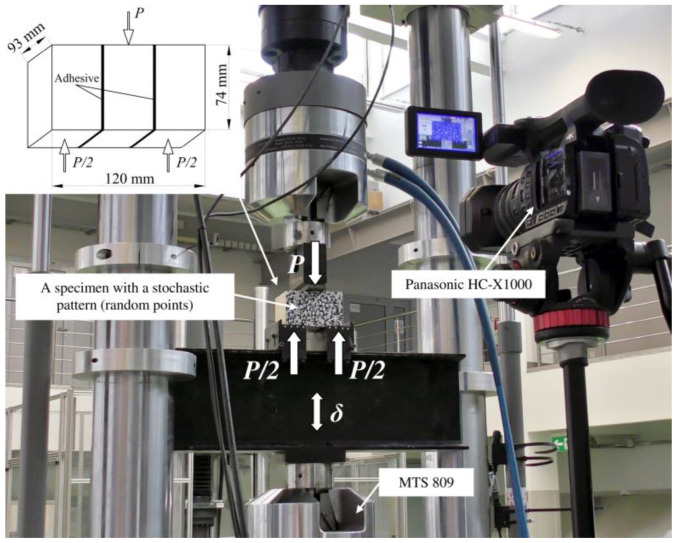
Laboratory setup of a double-lap shear test.

The “K” samples included three glued lamellas, while the “KW” samples had three lamellas and two CFRP strips. In both configurations, the analysis of the glue layer’s cohesive stiffness was based on the double-lap theoretical model presented by Tsai [56,57,58]. The relationship between a vertical force (*P*) and the relative displacement of the adherends (Δ*u*) is needed to use this solution. An MTS 809 testing machine registered the value of the force, while measuring the relative displacement was based on the procedures developed by the authors. Figure 10 shows the laboratory setup.

**Figure 11:**
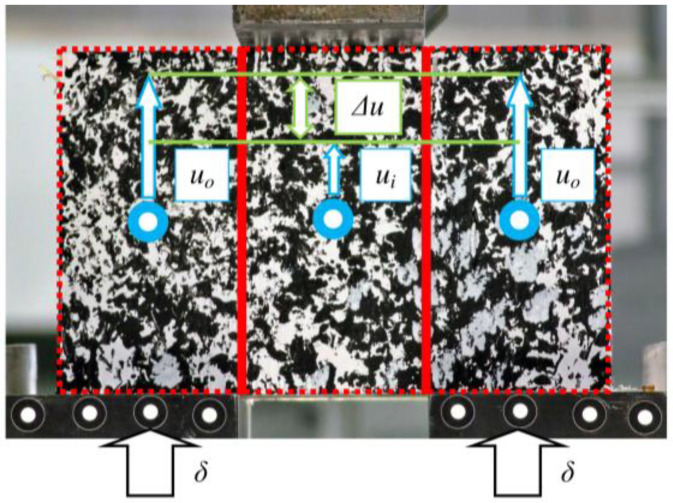
Denotations in a procedure for determining relative displacement (Δ*u*).

**Figure 12:**
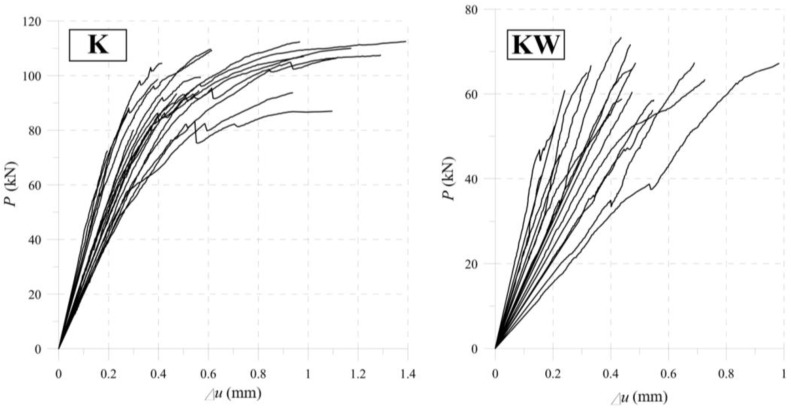
Laboratory tests results for the “K” and “KW” samples.

We measured displacements of both outer parts (*u_o_*) and the inner part (*u_i_*) of a specimen using a DIC (digital image correlation) method. A black and white stochastic pattern, otherwise called random points, allowed us to read the value of the displacement of every point in the sample. Selecting the centre of the adherends led to omitting the edge effects. It was important for interpreting the results using a theoretical model. Figure 11 shows the denotations described in the current paragraph, and Figure 12 shows the results of the experimental tests.

**Figure 13:**
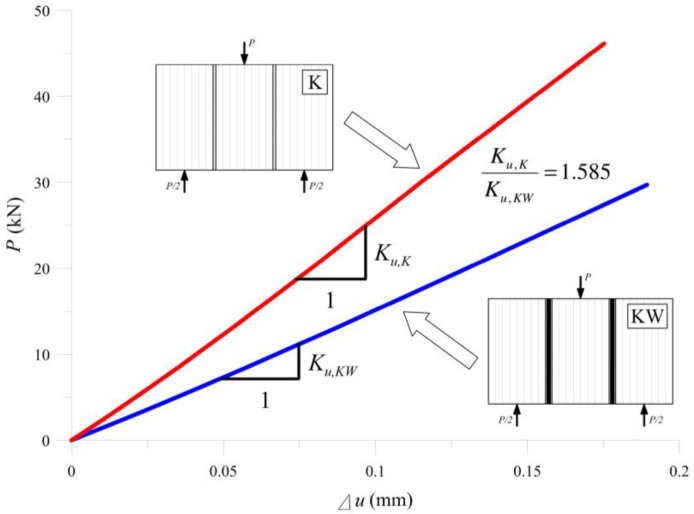
Stiffness measurement results for the samples “K” and “KW”.

It is worth noting that the average results for uncertainty is on the level of 23%—as is the uncertainty for wood members (approximately 20%). Figure 13 shows the elastic stiffness of the tested specimens. Defining the stiffness is equivalent to determining the average slope of the lines in the force range 5–20 kN.

**Figure 14:**
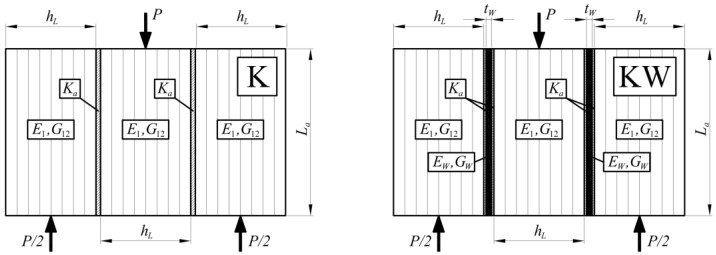
Denotations for the modified Tsai double-lab model (compare with Figure 10 and Figure 11).

To obtain the cohesive stiffness for the FE model, the authors proposed to use a theoretical double-lap shear solution [56,57,58]. Figure 14 presents the denotations for the variables used in the Formulas. The base assumption is introducing the cohesive stiffness (*K_a_*), substituting a dependency of a shear modulus (*G_a_*) on a thickness of the joint (*t_a_*):

(1) $$\frac{G_{a}}{t_{a}}=K_{a}$$

For this treatment, the value does not depend on the adhesive layer properties. This assumption is consistent with the intended use of the cohesive elements, designed to simulate an adhesive layer using FEM [59]. Following this topic, we can calculate the values for both wood–wood and wood–CFRP connections.

For the situation of the “K” sample, the total difference in displacements (Δ*u*) between connected elements is the sum of three consecutive components, relative displacement resulting from longitudinal deformation of the joined elements (Δ*u_ε_*), relative displacement resulting from the shear deformation of the connected elements (Δ*u_γ_*) and relative displacement resulting from the shear deformation of the adhesive layer (Δ*u_a_*):

(2) $$\Delta u_{K}=\Delta u_{a}+\Delta u_{\varepsilon }+\Delta u_{\gamma }$$

(3) $$\Delta u_{a}=\frac{1}{K_{a}}\frac{P}{2bL_{a}},\;\Delta u_{\varepsilon }=\frac{PL_{a}}{2bh_{L}E_{1}},\;\Delta u_{\gamma }=\frac{Ph_{L}}{4bL_{a}G_{12}}$$

Other variables that need to be described according to Figure 14 are: *b*—cross-sectional width, *h_L_*—height of a single lamella, *G*_12_—shear modulus of the wood and *L_a_*—vertical length of the lap joint.

By substituting all the formulas from Formula (3) to Formula (2), it is possible to find the displacement function depending on the cohesive stiffness (4):

(4) $$\Delta u_{K}(K_{a})=\frac{P}{2b}\left(\frac{1}{K_{a}L_{a}}+\frac{L_{a}}{h_{L}E_{1}}+\frac{h_{L}}{2L_{a}G_{12}}\right)$$

For the situation of the “KW” sample, for the differences in the model, we should add the shear deformations of the CFRP strips (Δ*u_W_*) and the shear deformations of the second adhesive (doubled value of Δ*u_a_*):

(5) $$\Delta u_{W}=\frac{Pt_{W}}{4bL_{a}G_{W}},\;\Delta u_{KW}=2\Delta u_{a}+\Delta u_{\varepsilon }+\Delta u_{\gamma }+\Delta u_{W}$$

Other variables that need a description according to Figure 14 are: *t_W_*—thickness of the CFRP tape and *G_W_*—shear modulus of the CFRP tape. Then the displacement function depending on the cohesive stiffness is:

(6) $$\Delta u_{KW}(K_{a})=\frac{P}{2b}\left(\frac{2}{K_{a}L_{a}}+\frac{L_{a}}{h_{L}E_{1}}+\frac{h_{L}}{2L_{a}G_{12}}+\frac{t_{W}}{2L_{a}G_{W}}\right)$$

The mean slope of the force–displacement (*P/*Δ*u*) curves in the linear–elastic range determines the stiffness of the entire setup in laboratory tests. Formula (7) gives the values of the stiffness and Figure 13 presents them as a chart.

(7) $$K_{u,K}=269.87\frac{kN}{mm},\;K_{u,KW}=170.29\frac{kN}{mm}$$

A simple recursive procedure leads to determining the cohesive stiffness, starting with one value of the force in a linear–elastic range, e.g., 20 kN. Next, it continues with calculating a relative displacement (Δ*u*) resulting from the laboratory tests and changing the cohesive stiffness (*K_a_*) in Formulas (4) and (6) up to get a good agreement with the theoretical model. Results for the “K” samples were:

(8) $$\Delta u_{K,lab}=\frac{P}{K_{u,K}}=\frac{20kN}{269.87\frac{kN}{mm}}=0.07411mm$$

(9) $$\Delta u_{K}\left(K_{a}\right)=0.07411mm\to K_{a}=91.32\frac{MPa}{mm}$$

Calculations of the “KW” samples’ cohesive stiffness resulted in:

(10) $$\Delta u_{KW,lab}=\frac{P}{K_{u,KW}}=\frac{20kN}{170.29\frac{kN}{mm}}=0.11745mm$$

(11) $$\Delta u_{KW}\left(K_{a}\right)=0.11745mm\to K_{a}=49.51\frac{MPa}{mm}$$

The wood–wood connection showed 85% higher stiffness than the wood–CFRP joint. It may lead to no-profits from using many CFRP strips in construction.

The last step in the analysis of a double-lap connection is preparing the FE model. For performing numerical analyses, we chose the professional Simulia ABAQUS 2019 software. In contrast to simplified models, the FEM enables to take into account the cohesive stiffness of a glue joint. Intended for this purpose are cohesive elements COH3D8 (8 nodes) [59], which stabilises solving from the very beginning. To model wood, we used C3D20 elements (27 nodes) as recommended by Hemanth [60] and tested by the authors of Reference [61]. The CSS8 elements (8 nodes), introduced by Vu-Quoc [62], represent CFRP strips and R3D4 elements described in ABAQUS documentation [63]—supporting plates.

**Figure 15:**
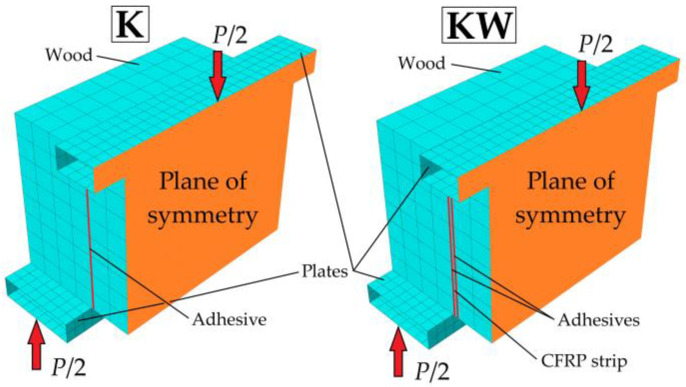
Finite element (FE) models of the adhesive connections.

Modelling only half of the symmetric geometry of the specimens stabilised and sped up the calculations (Figure 15). The mesh size was approximately 10 mm × 10 mm × 10 mm for the wood, 5 mm × 5 mm × 1.4 mm for the CFRP tapes, 5 mm × 5 mm × 0.1 mm for the adhesive layers and 5 mm × 5 mm for the steel plates. We took the wood properties from Table 2 in Section 4.

**Figure 16:**
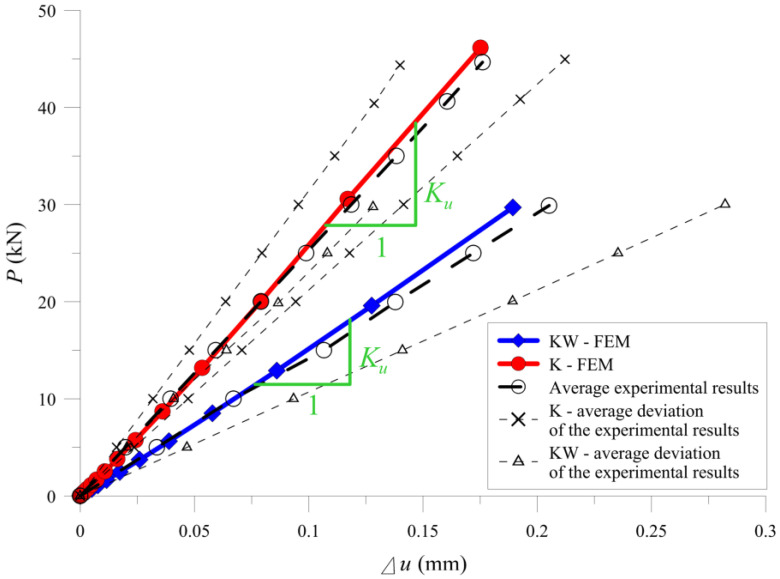
Comparison between the finite element method (FEM) and the laboratory results.

The cohesive stiffness calculated from Formulas (9) and (11), after applying to both models, gave very close results of the average stiffness of the setup compared to the experimental tests (Figure 16 and Table 1). It confirms the possibility for applying the Tsai model in determining the cohesive stiffness for the needs of FE model in the presented static scheme.

## 4. Laboratory Tests—Bending

The next study with the girder specimens was to measure the stiffness properties of the samples in a bending test. The experiments were displacement controlled (2 mm/min) and took place on the Zwick/Roell Z3000H (Zwick/Roell Poland Ltd., Lodz, Poland) testing machine with a constant span between supports, *L* = 1800 mm. The HBM WA/50 mm transducer (Hottinger Baldwin Messtechnik GmbH, Darmstadt, Germany), mounted at the middle bottom of the specimen, measured the deflection in a linear–elastic range (*w*).

**Figure 17:**
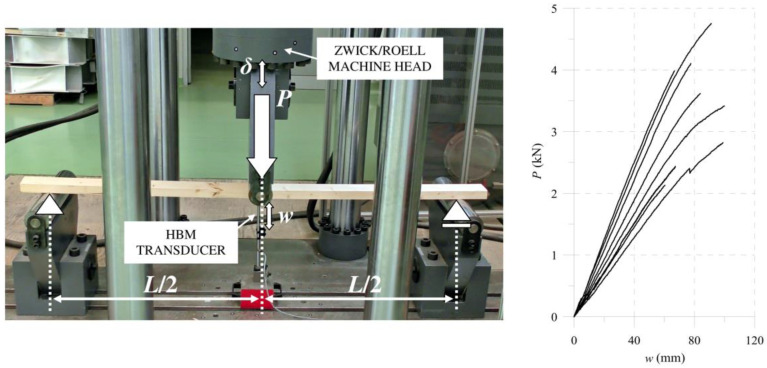
Laboratory setup and results.

At the beginning, we used a three-point bending scheme to test single wooden lamellas. It is mandatory to find basic wood properties. Figure 17 presents the laboratory setup and force-deflection (*P/w*) results derived from the machine and HBM transducer.

This allowed us to use the procedure developed by the authors in Reference [61], which is based on the average force–deflection relation (*P/w*). This relation is for an average slope of the curves in the elastic range as presented in Figure 17. Then, the longitudinal modulus of the elasticity were calculated from (12), where *b* and *h* are the width and height of the cross-section, respectively:

(12) $$E_{1}=\frac{P}{w}\left(\frac{L^{3}}{4bh^{3}}+\frac{24L}{5bh}\right)$$

The average value obtained from eight tested specimens, in dimensions of 93.4 mm × 40 mm × 2000 mm, was in the C24 class declared by the ABIES Poland Ltd. and was equal to *E*_1_ = 11.439 ± 2.288 GPa. The uncertainty of the measured parameter, as shown in the data in Figure 17, was approximately 20% around the mean value, typical for wooded elements. Then, the other necessary elastic properties resulted from the empirical dependencies. The authors present the procedure in one of their papers which includes modelling bent softwood elements [61]. We refer the readers to the mentioned article, available in open access. Table 2 presents the final average wood properties used in the further analyses.

**Figure 18:**
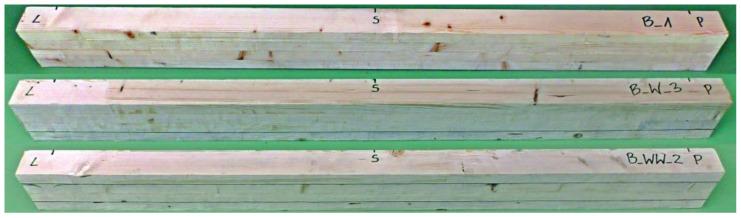
Exemplary “full-scale” girders after planing.

The test programme of full-scale girders (Figure 18) included:

- 7 samples made of glue laminated timber (B);
- 7 samples with one CFRP strip (BW);
- 7 samples with two CFRP strips (BWW).

Figure 9 in Section 2 presents the cross-sections in every mentioned configuration, and Table 3 collects the average dimensions.

**Figure 19:**
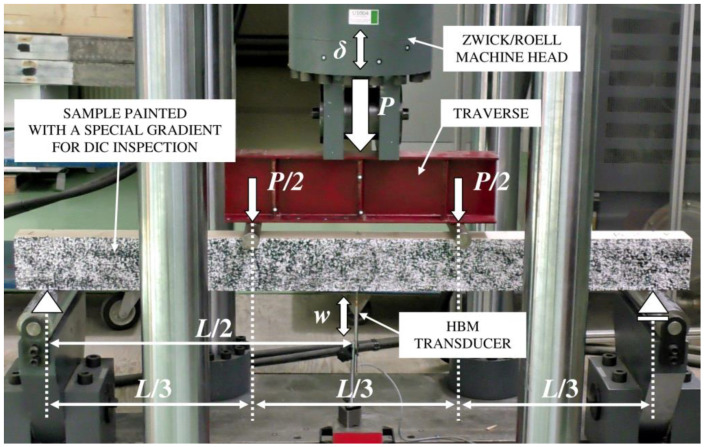
Laboratory setup.

**Figure 20:**
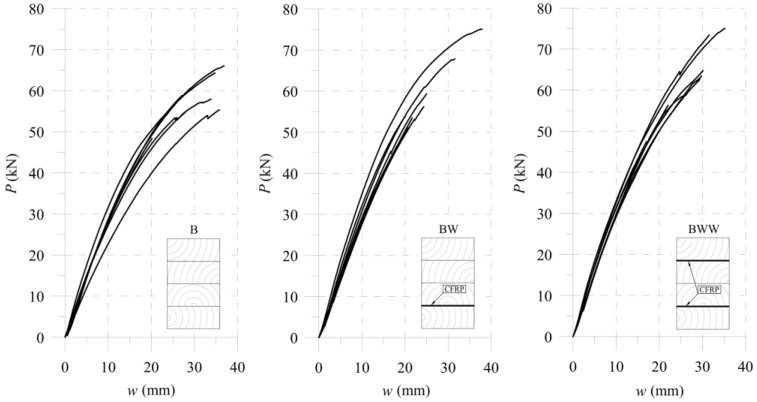
Laboratory tests results for full-scale girders.

A four-point bending test was used to measure the beams, requiring supplementary equipment such as I-beam parts with rollers. Figure 19 shows the laboratory setup for the test, and Figure 20 shows the curves from the laboratory tests. The bottom supports span was *L* = 1800 mm, and it was the same for every girder. In the used static scheme, the smaller concentrated forces occurred compared to a three-point bending test. It prevented wood from crushing near the loading rolls.

A perpendicular set of the Panasonic HC-X1000 4K camera (Kadoma, Osaka, Japan) enabled us to see the front surface between loading points. It allowed us to compare the various results for the specimens up to their fracture without exposing the sensors to damage.

## 5. Comparison between Simplified Model and FE Analysis

This section presents a simple model of the equivalent cross-sectional area used in a theory of composites [64,65,66]. The method consists of replacing the actual cross-section of a beam made of original materials with an equivalent cross-section and made with only one material of selected modulus of elasticity. The height remains unchanged by adjusting the width of another material. The most important simplification is assuming the connection between the materials to be perfect. It means neglecting the cohesive effects occurring in the adhesive. Considering an example using four-point bending, including a simplified correction for shear deformations, the general deflection Formula (13) is as follows (where *J* is a bending moment of inertia):

(13) $$w=\frac{23}{1296}\frac{PL^{3}}{EJ},\;E=0.9E_{1}$$

**Figure 21:**
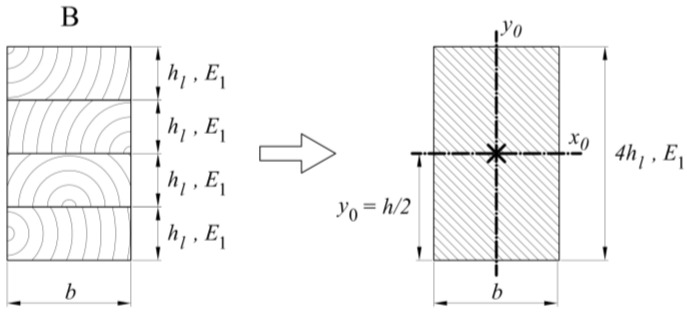
Cross-section of B-type samples, including the parameter notation used in the text.

The analyses include three types of beams, as shown in Section 2. Calculations for the B-type beams in bending reduce calculations for the full section (Figure 21), resulting in a moment of inertia for *J* such as in Formula (14).

(14) $$J=\frac{16bh_{L}{}^{3}}{3}$$

**Figure 22:**
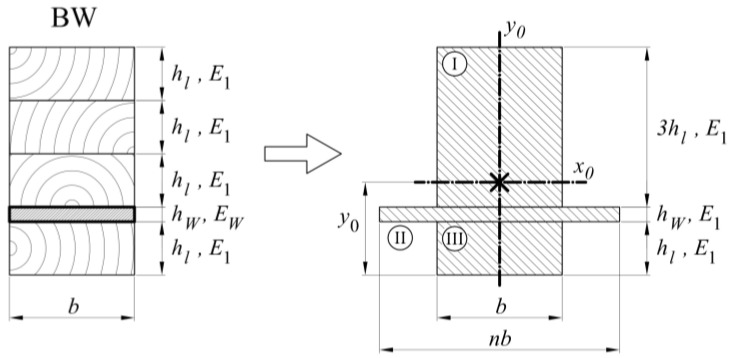
Cross-section of BW-type samples, including the parameter notation used in the text.

For BW-type beams (Figure 22), we replaced the section of laminate with the new width defined as wood. Then the Formulas (15)–(20) were given a moment of inertia value. Other variables used in the formulas were: *n*—coefficient determining a relation between elastic modules of CFRP tape and wood; *E_W_*—CFRP elastic modulus; *y*_0_—distance of the neutral axis from the bottom of the cross-section; *S_x_*—cross-sectional static moment; *A*—cross-sectional area; *J_I_*, *J_II_*, *J_III_*—components of the entire moment of inertia; *b*—width of a cross-section; *h_L_*—height of a single lamella, *t_W_*—thickness of a CFRP tape.

(15) $$n=\frac{E_{W}}{E_{1}},\;y_{0}=\frac{S_{x}}{A},\;J=J_{I}+J_{II}+J_{III}$$

(16) $$A=b\left(4h_{L}+nt_{W}\right)$$

(17) $$S_{x}=\frac{b}{2}\left(nt_{W}{}^{2}+16h_{L}{}^{2}+6h_{L}t_{W}+2nh_{L}t_{W}\right)$$

(18) $$J_{I}=\frac{9bh_{L}{}^{3}}{4}+3bh_{L}{\left(t_{W}+\frac{5}{2}h_{L}-y_{0}\right)}^{2}$$

(19) $$J_{II}=\frac{nbt_{W}{}^{3}}{12}+nbt_{W}{\left(y_{0}-h_{L}-\frac{1}{2}t_{W}\right)}^{2}$$

(20) $$J_{III}=\frac{bh_{L}{}^{3}}{12}+bh_{L}{\left(y_{0}-\frac{h_{L}}{2}\right)}^{2}$$

**Figure 23:**
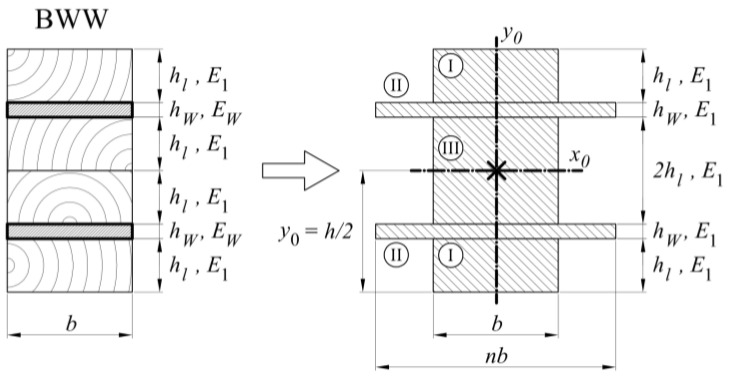
Cross-section of BWW-type samples including the parameter notation used in the text.

In the last case, the BWW-type beam, the calculations were simplified because the neutral axis remained in the centre of the cross-section (Figure 23) and the moment of inertia resulted from Formulas (21)–(24).

(21) $$n=\frac{E_{W}}{E_{1}},\;J=J_{I}+J_{II}+J_{III}$$

(22) $$J_{I}=\frac{bh_{L}{}^{3}}{6}+\frac{1}{2}bh_{L}{\left(2h_{L}+2t_{W}+h_{L}\right)}^{2}$$

(23) $$J_{II}=\frac{nbt_{W}{}^{3}}{6}+\frac{1}{2}nbt_{W}{\left(2h_{L}+t_{W}\right)}^{2}$$

(24) $$J_{III}=\frac{2bh_{L}{}^{3}}{3}$$

**Figure 24:**
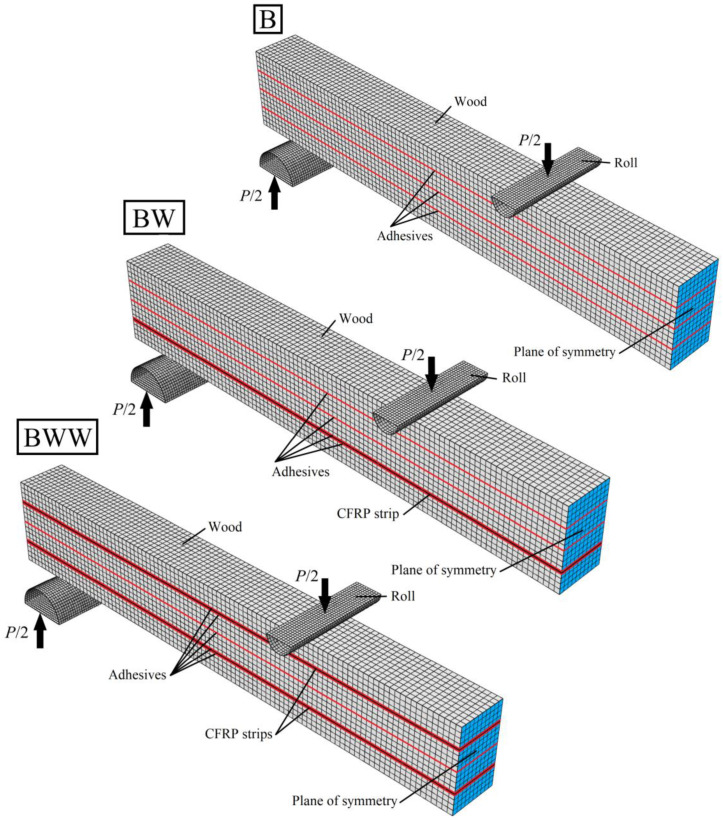
FE models for B, BW and BWW beams.

The earlier formulas allow for evaluating the load-deflection characteristics using the geometrical and material properties from Table 1, Table 2, Table 3 and Table 4, and to compare with the measured data in Figure 20. For better prediction of the beams’ behaviour, we prepared the FE models, taking into account the cohesive stiffness as determined in Section 3. Figure 24 presents the prepared FE solution. The parts have the same finite element types and dimensions as declared in Section 3. The plane of symmetry is in the sample’s centre.

We applied the wood properties from Table 2 in Section 4. It is important to distinguish between the cohesive stiffness of wood–wood (Formula (9)) and wood–CFRP (Formula (11)) joints as mentioned in Section 3. We modelled CFRP as anisotropic plates with the average properties defined based on producer technical information [67,68] as presented in Table 4. Performing calculations, both for FE and the equivalent area model, needs defining of the other parameters as single lamella thickness, *h_L_* = 40 mm, and the average beam width, *b* = 93.4 mm.

**Figure 25:**
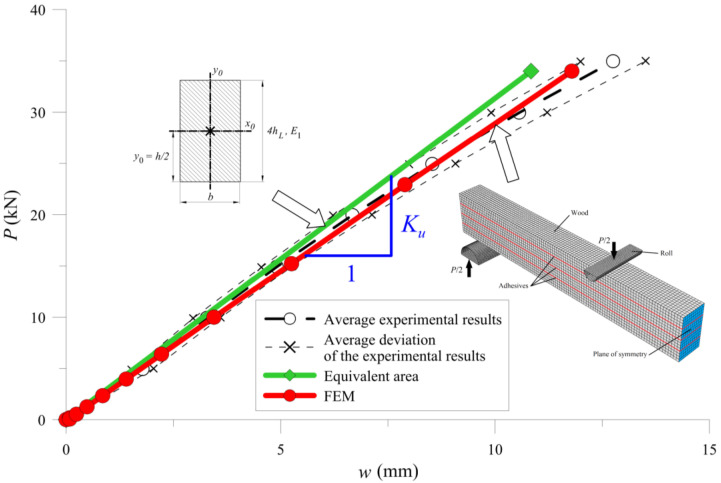
Results for B-type of the sample.

**Figure 26:**
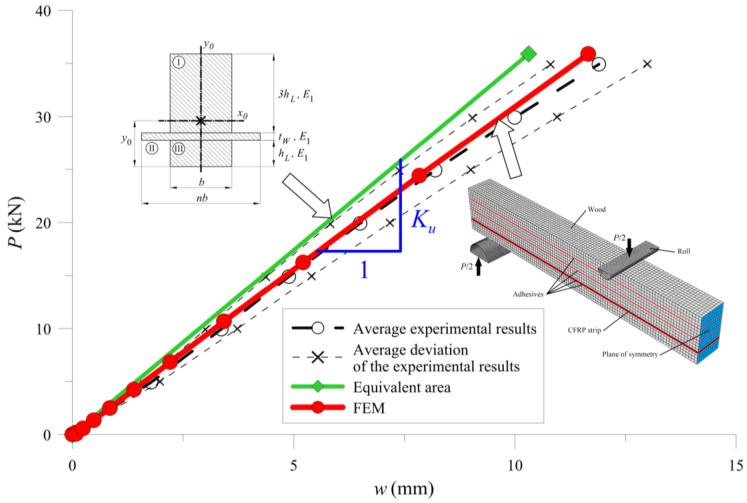
Results for BW-type of the sample.

**Figure 27:**
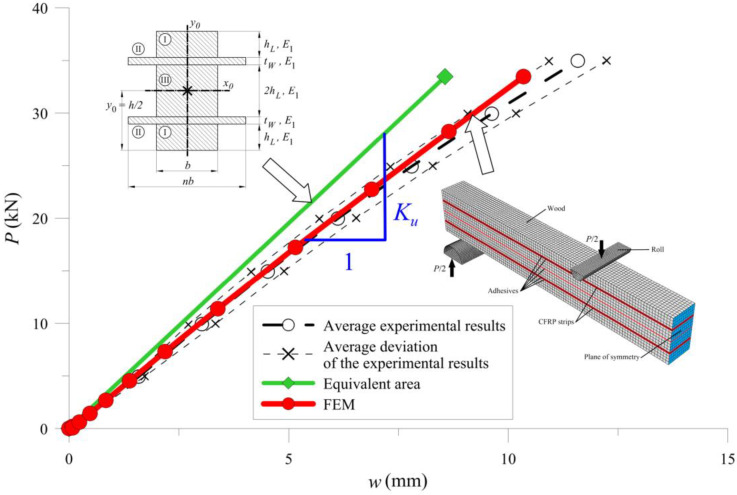
Results for BWW-type of the sample.

Figure 25, Figure 26 and Figure 27 show the bending test results obtained with the simplified model and the FE model compared to the measured data, represented as an average and average deviation (approximately 7% around the mean value) of the beams stiffness.

**Figure 28:**
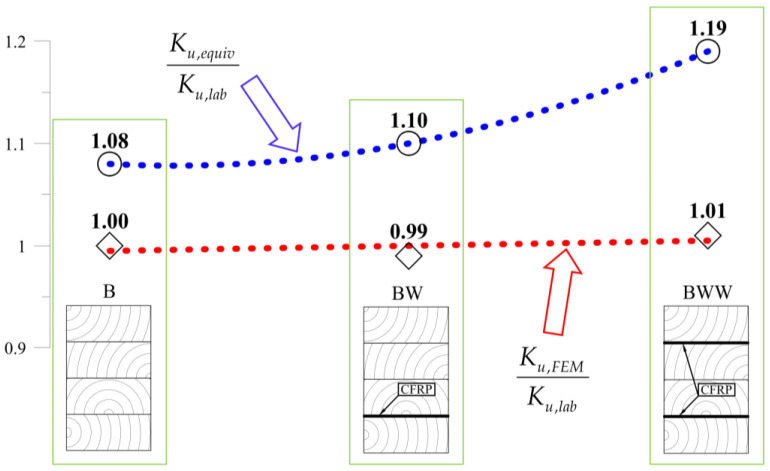
Comparison of the results for FE and equivalent cross-sectional model.

Compared with results from calculations from the experimental tests, one can see that the beam sustained over-stiffened behaviour in the case of using equivalent area theory. Adding more CFRP inserted into the cross-section increased the stiffness differences (Figure 28).

The analytical model assumed an ideal connection made by the joint (neglecting the joint itself). It was equivalent to a node-to-node joint in between materials in an FE model. Other researchers ignored joint stiffness during modelling wood–CFRP girders [35,45].

The FE model’s results were close to the mean measured values, showing that the method of selecting both the material (wood and CFRP) and the adhesive properties was correct. Table 5 and Figure 28 present a more detailed comparison of the stiffness results. Comparing the stiffness, one can notice that the FE model was near the laboratory values, while the equivalent cross-sectional model led to differences that grew in a parabolic manner.

## 6. Conclusions

The above experimental tests and theoretical calculations led to a very important conclusion. Neglecting the cohesive stiffness of adhesive layers, by considering the joints as perfect, may lead to an overestimation of the stiffness of the glued composite. It was significant with the wood–CFRP connection, which showed nearly twice as less stiffness than the wood–wood connection. The authors state that taking the cohesive stiffness of the adhesive into consideration is crucial in the case of strengthening wood with CFRP materials. 

The simplified models assuming a perfect connection between adherends are inappropriate for a PUR glue. According to this conclusion, the authors propose to use the FE method to take into account the cohesive stiffness. The use of properties obtained from the experiments and theoretical model of a double lap joint in the FE model has made it possible to predict the stiffness of the beams close to the experimental tests.

The research on wood–CFRP girders bonded on PUR glue is not thorough. According to this, many possibilities to develop the subject occur, it may be, for example, other reinforcement configurations, a study of the long-term load-bearing capacity of girders, an impact of variable loads or an influence of the different atmospheric conditions.

## Figures and Tables

**Table 1:** Comparison of the stiffness of the “K” and “KW” samples.

Beam Type	Average Stiffness of the “K” and “KW” Samples (kN/mm)		Comparison
	Experimental	FEM	$\frac{K_{u,FEM}}{K_{u,lab}}$
	***K_u,lab_***	***K_u,FEM_***	
“K”	269.87	270.36	1.00
“KW”	170.29	173.18	1.02

**Table 2:** Average wood properties in laboratory tests and Reference [61].

Description	Parameter	Value
Elastic modules in a three-dimensional state (GPa)	*E*_1_	11.439
	*E*_2_	0.732
	*E*_3_	0.458
Shear modulus in a three-dimensional state (GPa)	*G*_12_	0.715
	*G*_13_	0.529
	*G*_23_	0.069
Poisson coefficients (/)	*ν*_12_	0.335
	*ν*_13_	0.358
	*ν*_23_	0.416

**Table 3:** Average dimensions of the specimens. Notations according to Figure 9 from Section 2.

Configuration Type	Height (mm)	Width (mm)
B	159.4	93.4
BW	160.8	93.5
BWW	162.1	93.3

**Table 4:** Average properties of CFRP tapes according to References [67,68].

Description	Parameter	Value
Elastic modulus of S&P C-Laminate (GPa)	*E*_1_ = *E_W_*	175
Elastic modulus of Resin 220 (GPa)	*E*_2_ = *E*_3_	7.10
Shear modulus of epoxy resin (GPa)	*G*_12_ = *G*_13_ = *G*_23_ = *G_W_*	2.73
Poisson coefficients (/)	*ν*_12_ = *ν*_13_ = *ν*_23_	0.3
CFRP strip thickness (mm)	*t_W_*	1.4

**Table 5:** Comparison of the beams’ stiffness.

Beam Type	Average Stiffness of the Beams (kN/mm)			Comparison	
	Experimental	Equivalent Area	FEM	$\frac{K_{u,equiv}}{K_{u,lab}}$	$\frac{K_{u,FEM}}{K_{u,lab}}$
	***K_u,lab_***	***K_u,equiv_***	***K_u,FEM_***		
B	2.913	3.136	2.915	1.08	1.00
BW	3.165	3.482	3.129	1.10	0.99
BWW	3.278	3.907	3.307	1.19	1.01
